# Preventing anxiety problems in children with Cool Little Kids Online: study protocol for a randomised controlled trial

**DOI:** 10.1186/s13063-015-1022-5

**Published:** 2015-11-05

**Authors:** Amy J. Morgan, Ronald M. Rapee, Elli Tamir, Nahal Goharpey, Agus Salim, Lauren F. McLellan, Jordana K. Bayer

**Affiliations:** School of Psychology and Public Health, La Trobe University, Kingsbury Drive, Bundoora, Victoria 3086 Australia; Centre for Emotional Health, Building C3A, Level 7, Macquarie University, Sydney, NSW 2109 Australia; Department of Mathematics and Statistics, School of Engineering and Mathematical Sciences, La Trobe University, Kingsbury Drive, Bundoora, Victoria 3086 Australia; Murdoch Childrens Research Institute, Royal Children’s Hospital, Flemington Road, Parkville, Victoria 3052 Australia; Department of Paediatrics, University of Melbourne, Royal Children’s Hospital, Flemington Road, Parkville, Victoria 3010 Australia

**Keywords:** Anxiety disorders, Prevention, Internet, Parent training, Inhibition, Young children

## Abstract

**Background:**

Anxiety disorders are the most common type of mental health problem and begin early in life. Early intervention to prevent anxiety problems in young children who are at risk has the potential for long-term impact. The ‘Cool Little Kids’ parenting group program was previously established to prevent anxiety disorders in young children at risk because of inhibited temperament. This group program was efficacious in two randomised controlled trials and has recently been adapted into an online format. ‘Cool Little Kids Online’ was developed to widen and facilitate access to the group program’s preventive content. A pilot evaluation of the online program demonstrated its perceived utility and acceptability among parents. This study aims to evaluate the efficacy of Cool Little Kids Online in a large randomised controlled trial.

**Methods/Design:**

Parents of young children who are 3–6 years old and who have an inhibited temperament will be recruited (n = 385) and randomly assigned to either immediate access to Cool Little Kids Online or delayed access after a waiting period of 24 weeks. The online program contains eight modules that help parents address key issues in the development of anxiety problems in inhibited children, including children’s avoidant coping styles, overprotective parenting behaviours, and parents’ own fears and worries. Intervention participants will be offered clinician support when requested. The primary outcome will be change in parent-reported child anxiety symptoms. Secondary outcomes will be child internalising symptoms, child and family life interference due to anxiety, over-involved/protective parenting, plus child anxiety diagnoses assessed by using a new online diagnostic tool. Assessments will take place at baseline and 12 and 24 weeks after baseline.

**Discussion:**

This trial expands upon previous research on the Cool Little Kids parenting group program and will evaluate the efficacy of online delivery. Online delivery of the program could result in an easily accessible evidence-based resource to help families with young children at temperamental risk for anxiety disorders.

**Trial registration:**

Australian New Zealand Clinical Trials Registry 12615000217505 (registered 5 March 2015)

**Electronic supplementary material:**

The online version of this article (doi:10.1186/s13063-015-1022-5) contains supplementary material, which is available to authorized users.

## Background

Anxiety disorders are the most common type of mental health problem in children and adolescents and occur early in development [1]. Childhood anxiety disorders cause substantial impairment in family functioning, peer and social activities, and functioning at school [2–4]. Untreated anxiety disorders appear to have a chronic course, and longitudinal research indicates significant links between anxiety in childhood, adolescence and adulthood [5–7]. Childhood anxiety is also associated with lower educational attainment [8], lower earnings in adulthood [9], and greater risk of developing other mental health problems such as depression and substance misuse [5, 10]. Given the low rates of treatment utilisation [11] and often long delays before seeking help [12], there is a clear rationale to intervene early with children at risk.

The causes of anxiety disorders are complex and interactive; however, several risk factors in children have been identified as being important. These include an inhibited temperament, overprotective or overcontrolling parenting behaviours, negative or critical parenting, and parent modelling of anxious thoughts and behaviour [13, 14]. Interventions have begun to be developed and evaluated for their potential in preventing anxiety disorders in children selected for being at-risk [15]. One such preventive intervention is a parenting group program called Cool Little Kids [16]. This parent education program aims to help young children who have an inhibited temperament, which is a key risk factor for anxiety disorders and can be identified at a young age. The Cool Little Kids program addresses significant factors in the development of anxiety problems in inhibited young children, including children’s avoidant coping styles, overprotective parenting behaviours, and parents’ own fears and worries. The program has been shown in two randomised controlled trials (RCTs) [16, 17] to prevent child anxiety disorders and is undergoing further evaluation using a population screening model [18]. One RCT evaluated the preventive effects of the intervention 1, 2 and 3 years later and found significantly fewer anxiety disorders in the intervention group than the control group, with intervention effects becoming stronger over time [16, 19]. A recent long-term follow-up conducted when the children had entered the high-risk period of adolescence showed that intervention effects had persisted for girls [20]. The population cost-effectiveness of this intervention has also been evaluated and was found to represent very good value for money [21]. The Cool Little Kids parenting group program therefore has a strong evidence-base supporting its aim of preventing anxiety disorders in children.

The Cool Little Kids parenting group program was designed to be as brief as possible in order to maximise its potential for public health use; however, barriers to its wider dissemination in the community remain. There are workforce issues associated with the scarcity of trained psychologists available to widely deliver the program. Parents with young families also face substantial barriers to attending group programs, including time demands and scheduling issues, as well as practical barriers such as transportation and arranging child care [22]. Making preventive programs easily accessible is key to engaging and retaining parents. The internet offers opportunities to overcome some of these barriers and hence delivering parenting programs over the internet could potentially reach a greater number of parents more cost-effectively. Recognising this potential, existing parenting programs for early-onset child externalising problems have been successfully adapted to an online format [23]. Similarly, the Cool Little Kids parenting group program was developed into an online format, ‘Cool Little Kids Online’, to widen and facilitate access for parents to the group program’s preventive content. Parents can use the online program from the convenience of their own home at any time of day. The program can also reach parents in regional and remote areas with little access to mental health services.

Cool Little Kids Online has been piloted with a sample of 51 parents to explore its acceptability and perceived utility in reducing anxiety in temperamentally inhibited young children (Morgan AJ, Rapee RM, Bayer JK: Prevention and early intervention of anxiety problems in young children: A pilot evaluation of Cool Little Kids Online, submitted). This study compared two options for delivering the program online: a supported version in which parents received scheduled calls from a clinician and a purely self-help version with no clinician support. Parents could access the program for 10 weeks and provided data at baseline and post-intervention. Results from the pilot study were encouraging, as parents reported high levels of satisfaction with the program and there were significant improvements in child anxiety. The program has since been refined and improved on the basis of pilot study feedback and now merits evaluation in a full-scale RCT. The aim of the current study is therefore to evaluate the efficacy of Cool Little Kids Online in children at temperamental risk of anxiety disorders. We hypothesise that, compared with a waitlist control, the program will lead to lower child anxiety and internalising symptoms, lower life interference related to anxiety, lower scores on overprotective parenting, and fewer child anxiety disorders. We anticipate that the program will be rated as useful by parents. In addition to exploring these hypotheses, we will explore for whom the program is most effective (e.g., child and family demographics and child anxiety type).

## Methods/Design

### Design

This study is an RCT of the Cool Little Kids Online program. Participants will be randomly allocated to either the intervention group or a delayed access (waitlist) group. The intervention group will receive immediate access to the Cool Little Kids Online program, whereas participants allocated to the waitlist group will receive access after a delay of 24 weeks. Because the Cool Little Kids parenting group program has been shown to be efficacious, it was deemed unethical to withhold intervention entirely. At least one previous trial has shown a difference in efficacy that emerged by 6 months [17] and therefore a 24-week waitlist was selected as a good balance between offering some form of help to all participants and controlling for threats to internal validity. The study was approved by the La Trobe University Human Ethics Committee (UHEC 15–010) and is registered in the Australian and New Zealand Clinical Trials Registry (12615000217505). See Additional file 1 for the completed SPIRIT (Standard Protocol Items: Recommendations for Interventional Trials) checklist of recommended items to address in a clinical trial protocol.

### Participants and procedure

#### Inclusion and exclusion criteria

Participants will be eligible for the trial if they are a parent of a 3- to 6-year-old child with an inhibited temperament. Inhibition will be indicated by a score of more than 30 on the Short Temperament Scale for Children (STSC) – Approach subscale [24, 25]. This measure was used to identify children at temperamental risk of anxiety problems in previous research on the Cool Little Kids program [16, 18]. A score of more than 30 indicates behavioural inhibition at or above the 85th percentile. Parents will also need to have internet access to use the online program. Parents who report that their child has cerebral palsy, an intellectual disability, or severe autism will be ineligible, as the Cool Little Kids program as a whole is not suitable for children with severe developmental problems. Parents who do not reside in Australia will not be eligible to participate, because telephone support is offered to parents in the intervention group.

#### Recruitment

Participants will be recruited via the Cool Little Kids Online website [26], and all promotional materials will direct potential participants to this website. The website will be promoted with advertisements on Facebook, Google, and other websites as well as via preschool services.

#### Enrolment and randomisation

Potential participants will be screened online for eligibility and, if eligible, will provide online informed consent. Parents who consent to participate then complete the online baseline questionnaire. Randomisation to condition will occur at the end of the baseline questionnaire, when the participant is considered enrolled. A computer script will automatically randomly allocate parents to study arms in a 1:1 allocation (simple randomisation). Allocation concealment will thus be ensured, as neither the participant nor study investigators will be able to predict the allocation. Parents will be informed about their allocation in an email and will also be telephoned to welcome them to the study within the first week of their participation. Parents in the intervention condition will be emailed instructions on how to log in to the online program once they have been enrolled. Parents in the delayed-access condition will be emailed these instructions once they have completed the third questionnaire after a waiting period of 24-weeks. Owing to the nature of the intervention, participants and study investigators cannot be blinded to allocation.

### Intervention

The Cool Little Kids parenting group program targets key factors related to the development of child anxiety disorders. It teaches parents practical ways to reduce child anxiety and fears through graded exposure, contingency management, reducing overprotective behaviours, and managing parents’ own fears and worries. Cool Little Kids Online was directly adapted from the Cool Little Kids parenting group program and has eight interactive online modules containing a mix of written information, videos, audio narration, interactive worksheets and activities, and parent experiential stories. See Table 1 for a content overview of each module. A new module becomes available each week in sequential order. Parents are encouraged to complete one module per week as well as home practice activities with their child in between modules (e.g., exposure tasks). Modules consist of 26 web pages on average and take approximately 30–60 minutes to complete. Module text is written in short, simple sentences with an average Flesch-Kincaid Grade Level of 6.9, indicating suitability for individuals with a seventh grade education.

**Table 1:** Module content

Module number and title	Content overview
1. Understanding anxiety	• Nature of child anxiety, its development and the role of temperament
	• Overview of program content
	• Setting goals
2. Introducing stepladders	• Principles and application of exposure hierarchies (stepladders)
3. Using rewards	• Principles of using rewards effectively to reinforce child behaviour
4. Parenting an anxious child	• Role of overprotection in child anxiety
	• Alternative parental strategies, including encouraging greater child independence
2. Troubleshooting stepladders	• Review of stepladder progress
	• Troubleshooting difficulties that commonly occur
3. Overcoming barriers	• Overcoming barriers to stepladder practice
	• Introduction to cognitive restructuring for parents’ own worries
4. Managing worries	• Cognitive restructuring for parent worries, particularly related to implementing exposure with their child
	• Review of stepladders
5. Planning for the future	• Review of progress so far
	• Planning of strategies to use for future challenges or high-risk times such as starting school

The online format was developed with input from clients of the Emotional Health Clinic, Macquarie University, and feedback from participants in the population trial of Cool Little Kids conducted in Melbourne, Australia [18]. Parents who had participated in the group program delivered by psychology students at La Trobe University contributed their stories and experiences. The program’s online development was informed by research on persuasive design elements that maximise adherence [27, 28] and research on features that encourage the therapeutic alliance in internet-based interventions [29]. Participants are ‘guided’ through each module by a female coach, who ‘speaks’ to the participants in text speech bubbles or through voiceovers to animated videos and screen captures of how to complete worksheets. The coach approximates the role of the facilitator of the group program and aims to give the program a social presence, which is thought to enhance online program engagement by mimicking human-to-human interaction [28]. The program incorporates recommended principles of good e-learning instruction [30–32]. For example, the techniques of how to develop an exposure hierarchy and use realistic thinking are taught with several worked examples that fade out the amount of help provided to finish the example task. Difficult concepts are presented with a mix of video, images, and text, and modules combine new content with a review and practice of previous skills. Parents can self-monitor their progress by writing in an online diary as well as view a chart of their child’s fear symptoms over time, which they are prompted to rate at weekly intervals.

The online program uses a technological platform which was previously developed at Macquarie University for adolescents with anxiety problems and which was built upon the Symfony framework. The website is designed with a responsive layout so that it can be used on smaller devices such as tablets and smartphones. Usability testing was conducted with five parents of young children by using the ‘think aloud’ protocol [33] to improve the navigation and content presentation. The program was further improved and refined on the basis of feedback from parents who participated in the pilot study.

Several strategies were adopted to encourage parents to use the program and practice program skills. Participants receive automated emails after completing each module, which reinforce the home practice activities. Modules are considered completed when 80 % of pages, including the final summary page, have been viewed. Automated emails also announce the availability of each new module and serve as a reminder to use the program. Internet interventions that have frequent prompts and that have new content available on a regular basis are associated with greater use [34, 35]. Booster reminder emails are sent after 18 weeks and 1 week before parents’ 6-month access to the program ends. Participants in the intervention group will be sent one text message reminder after 2 weeks of website inactivity to check whether they are having technical problems and to encourage them to log in.

Parents in the intervention group will also be offered telephone support from a psychologist when requested. Support will be provided ‘on-demand’ rather than at scheduled intervals. The pilot study results suggested that some parents do not require extra support, and prior research has shown that support on-demand can still be effective [36, 37]. Support will assist parents to troubleshoot difficulties implementing intervention techniques. It will be provided by a provisionally registered psychologist with prior experience delivering the Cool Little Kids parenting group program, supervised by an experienced clinical psychologist. The psychologist will be able to examine parents’ completed worksheets and their progress through the online program in order to support them.

### Assessment

Parents will complete assessments online by using secure survey software at three time points: T1 (baseline), T2 (12 weeks after baseline), and T3 (24 weeks after baseline). See Table 2 for an overview of measures at each time point.

**Table 2:** Overview of measurements

Outcome	Scale	T0	T1	T2	T3
Temperamental inhibition	STSC-Approach subscale	✓			
Screening questions for eligibility	Child age category, child disability, printer access	✓			
Socio-demographics	Developed by authors		✓		
Child anxiety symptoms	PAS-R		✓	✓	✓
Child anxiety disorders	OAPA				✓
Child internalising symptoms	SDQ-ES		✓	✓	
Child internalising and externalising symptoms	SDQ				✓
Life interference from anxiety	CALIS-PV		✓	✓	✓
Over-involved/protective parenting	OI/P		✓		✓
Parent psychological distress	K10		✓		
Program evaluation (intervention arm only)	Developed by authors			✓	
Help-seeking	Developed by authors			✓	✓

T0 = screening, T1 = baseline assessment, T2 = 12 weeks after baseline, T3 = 24 weeks after baseline, *STSC* Short Temperament Scale for Children, *PAS-R* Revised Preschool Anxiety Scale, *OAPA* Online Assessment of Preschool Anxiety, *SDQ-ES* Strengths and Difficulties Questionnaire-Emotional Symptoms subscale, *SDQ* Strengths and Difficulties Questionnaire, *CALIS-PV* Children’s Anxiety Life Interference Scale – Preschool Version, *OI/P* Over-Involved/Protective parenting scale, *K10* Kessler 10 Psychological Distress Scale

#### Primary outcome

The primary outcome will be parent-reported child anxiety symptoms measured with the Revised Preschool Anxiety Scale (PAS-R) [38]. The PAS-R assesses anxiety symptoms in young children across four subscales: generalised anxiety, social phobia, separation anxiety, and specific phobias. It is an update of the Preschool Anxiety Scale [39], which was originally adapted from the Spence Children’s Anxiety Scale. In its recent revision, the scale dropped two items related to obsessive-compulsive disorder symptoms that were not reliable, leaving 28 items. The measure has good evidence supporting construct validity, and the total score and subscales have good internal consistency (α = 0.72–0.92) [38]. Total scores range from 0 to 112; a previous report in a large community sample of 3- to 5-year-olds suggests that the community mean score is 38 and the mean score in children with an anxiety disorder is 61 [38].

#### Secondary outcomes

##### Anxiety diagnoses

Child anxiety diagnoses will be assessed with a new measure called the Online Assessment of Preschool Anxiety (OAPA). Anxiety diagnoses might usually be considered a primary outcome of interest, but owing to the preliminary nature of the OAPA measure, anxiety diagnoses were included as a secondary outcome for this trial. The OAPA is an online assessment of anxiety diagnoses in young children (6 years or below), is based on Diagnostic and Statistical Manual of Mental Disorders, 4th Edition (DSM-IV) criteria, and is completed by parents. This diagnostic measure was adapted from the Online Assessment of Anxiety - Parent version (unpublished) developed by Lyneham and colleagues from Macquarie University. Parents are asked screening questions for child anxiety disorders (separation anxiety disorder, social phobia, generalised anxiety disorder, specific phobia), and automated rules determine whether the rest of the questions for that section are presented. For each anxiety problem, parents rate child anxiety symptoms and level of interference in closed questions. Parents are also asked to describe their child’s behaviours and thoughts related to each anxiety problem and write examples of anxiety-related life interference. Responses are automatically scored for the presence or absence of a disorder on the basis of DSM-IV criteria and then all responses undergo a clinical review by a psychologist. This review checks whether parents’ written descriptions are consistent with the disorder being assessed and whether the level of impairment described is clinically sufficient to warrant a diagnosis. Reviews will be performed blinded to group allocation.

##### Child internalising symptoms

Child internalising symptoms will be assessed with the Strengths and Difficulties Questionnaire-Emotional Symptoms subscale (SDQ-ES) [40]. The SDQ is a widely used screening tool for psychosocial problems in children. The parent report version for children from 4 to 10 years old has five items that comprise the Emotional Symptoms subscale, and scores range between 0 and 10. Psychometric properties of the SDQ-ES are adequate [41], and it has been shown to be sensitive to change from child anxiety treatment [42, 43].

##### Life interference

Life interference from child anxiety will be assessed with the Children’s Anxiety Life Interference Scale – Preschool Version (CALIS-PV) [44]. The CALIS assesses the impact of children’s anxiety symptoms on their own life and their family’s functioning. The CALIS-PV was adapted by Kennedy et al. [17] for use with preschool-age children. It is a 20-item parent-reported questionnaire with two subscales: child life interference from anxiety and family interference due to child anxiety. The internal consistency of CALIS-PV is excellent, and the total score is sensitive to change with treatment [17].

##### Over-involved/protective parenting

Over-involved/protective parenting practices will be assessed with the Over-Involved/Protective parenting scale (OI/P) [45]. The OI/P is an eight-item measure of parenting behaviours that discourage autonomy in young children (e.g., ‘I prevent my child getting involved in activities or tasks that he/she finds too difficult and may fail at’). Items are rated on a 4-point response scale and refer to specific behaviours rather than broad parenting statements to minimise social desirability bias. A 5-point version of the scale had good internal consistency (α = 0.81) and was significantly associated with child internalising symptoms [46].

#### Other measures

##### Socio-demographics

Socio-demographics will be assessed with questions on child sex, child date of birth, number of siblings and birth order, parent age, parent education level, marital status, language mainly spoken at home, household income, and weekly internet usage. Questions will also assess recruitment source, what parents hope to gain from the program, and what help they have already sought for their child’s shyness, fears or anxiety.

##### Parent psychological distress

Psychological distress in the parent will be assessed with the Kessler 10 Psychological Distress Scale (K10) questionnaire at baseline [47]. The K10 is a widely used measure and assesses 10 symptoms of mental health in the anxiety-depression spectrum. It has good internal consistency [47] and is a good discriminator between community ‘cases’ and ‘non-cases’ [48, 49]. According to Australian norms, scores of 10–15 indicate low, 16–21 moderate, 22–29 high, and 30–50 very high psychological distress [50].

##### Help-seeking

Parents are asked whether they have visited a health professional to help with their child’s shyness, fears or anxiety during the previous 3 months.

##### Program evaluation

A mix of open-ended and Likert-scale questions, adapted from previous research on the Cool Little Kids program, will be used to measure participant satisfaction and program feedback [51]. Five questions assess the usefulness of the program for learning about anxiety and how to manage it in their child, measured on a 5-point scale: ‘not at all’, ‘a little’, ‘quite’, ‘very’, or ‘extremely’ useful. Three open-ended questions ask parents for the best and worst aspects of the program and what would make it better. A 5-point scale assesses whether the parent would recommend the program to others: ‘definitely would’, ‘probably would’, ‘not sure’, ‘probably would not’, or ‘definitely would not’. Frequency of practice of program skills is assessed on a 4-point scale: ‘every day’, ‘a few times a week’, ‘once a week’, or ‘less than once a week’. Finally, three questions ask about the support telephone calls provided by the study’s clinician and their helpfulness.

##### Program use

Intervention use will be assessed by examining server records of number of logins, number of modules accessed, number of modules completed, and time spent logged in. Parents who complete fewer than eight modules will be asked about the role of six factors in not completing the program (‘not enough time’, ‘child improved and no longer needed help’, ‘sought help from a professional instead’, ‘program wasn’t helping’, ‘technical problems’, or ‘other’), measured on a 3-point scale: ‘no part’, ‘a little part’, or ‘a major part’*.*

##### Negative effects

Negative effects will be measured through symptom deterioration on the PAS-R (total scale). Children will be classified as deteriorated when they show a statistically reliable negative change between T1 and T3, according to the Reliable Change Index [52].

### Response rate sub-study

This RCT will explore the impact on responses rates of a prize draw for participants who complete the longer T3 questionnaire. Although incentives can increase response rates to cross-sectional surveys [53], there is surprisingly little evidence that providing monetary incentives increases response rates to follow-up questionnaires in intervention research [54, 55]. Therefore, participants will be randomly allocated to one of two groups: (1) the ‘informed’ group, who will be informed that they will enter a prize draw if they complete the T3 questionnaire, and (2) the control group, who will not be informed about the prize draw but will still be eligible to win. Participants in the ‘informed’ group will be informed of the prize draw in the email inviting them to complete the T3 questionnaire and at the start of this questionnaire. Randomisation to group will occur by automated computer script at the end of the baseline questionnaire, stratified by intervention (immediate access) and control (delayed access) conditions. Participants will have a 1 in 20 chance of winning a gift voucher worth AUD$50. Prize draw winners will be randomly selected via automated computer script at the end of the T3 questionnaire.

### Sample size calculation

The study will aim to recruit about 385 participants. This sample size gives 80 % power at the 5 % level of significance to detect an effect size of 0.32 standard deviations between conditions on the primary outcome measure, given a correlation of 0.5 between baseline and 24-week scores [56]. This effect size is the mean found in interventions designed to prevent anxiety in children and adolescents [57], and this sample size allows for an attrition rate of approximately 20 % at the final follow-up.

### Statistical analyses

Mixed-models analyses of variance will be conducted on all continuous outcomes to evaluate the effect of the intervention. The mixed-models approach is consistent with intention-to-treat analytic approaches under the assumption that data are missing at random. Any participants who are randomly assigned but withdraw from the study, or do not complete the intervention, will be included in these analyses as randomly assigned. Contrasts will compare change from baseline at the 12- and 24-week follow-ups between conditions as well as change between the 12- and 24-week follow-ups. Between-group effect sizes (Cohen’s *d*) will be calculated by standardising the differences between baseline and follow-up scores by the pooled standard deviation of the baseline scores. Responders will be identified as participants who demonstrate a statistically reliable improvement in anxiety symptoms on the PAS-R between baseline and T3, according to the procedures in Jacobson and Truax [52]. Response rates will be compared across conditions, and relative risk, the ratio of the probability of a response occurring in the intervention group versus the control group, will be calculated and tested for significance. The number needed to treat to achieve a response will be calculated with 95 % confidence intervals by using the method proposed by Bender [58].

Predictors of outcome will also be explored in addition to group allocation, including parent and child factors. The amount of program usage by intervention participants will be described, and predictors of program use will be explored. In addition to the intention-to-treat analyses, ‘per-protocol’ analyses will be conducted to examine the program’s effect in participants who have received an adequate ‘dose’ of the intervention. These exploratory analyses will evaluate the effectiveness of completing different amounts of the program (e.g., at least six modules). Statistical analyses will be conducted by using SPSS (IBM Corp, Armonk, NY, USA) and STATA (StataCorp, College station, TX, USA), and significance level will be set at a *P* value of less than 0.05.

## Discussion

This study will provide important information about the efficacy of an online parenting program that aims to prevent anxiety problems in young children. It will build on previous efficacy [16] and translational [18] research into the Cool Little Kids parenting group program by broadening the program’s potential reach to an online audience. Internet-based delivery of the program could result in an easily accessible evidence-based resource to help families with young children at temperamental risk for anxiety disorders.

This study extends the small feasibility pilot of Cool Little Kids Online to a large RCT with a longer follow-up of participants. It builds on the pilot study by enhancing program aspects thought to improve adherence to online interventions. The RCT tests a different model of providing clinician support ‘on-demand’, which maximises the efficient use of study resources whilst acknowledging the positive effects on adherence and outcomes that clinician support can achieve [59]. Support on-demand balances the need for a low-cost solution that can be sustainable into the future with the expected benefits that support can provide to parents who especially need it. This RCT also innovatively tests the effects of a prize draw on reducing attrition at the final assessment point. This recognises that although conducting the study online can make it easier to recruit participants and reach the target sample size, greater ease in joining an online study can also increase the likelihood that some participants will drop out, resulting in higher rates of attrition than a non-online trial [60].

Prevention and early intervention approaches to anxiety disorders are important, as treatment alone cannot avert the entire population disease burden [61]. Parenting programs to prevent internalising problems in young children are relatively rare, even though corresponding parenting programs for externalising problems are established as effective [62]. To our knowledge, this is the first RCT of an online selective intervention aimed at preventing anxiety problems in young children.

## Trial status

The study began in June 2015, and 270 participants had been recruited at the time of submission (21 Aug. 2015).

## Additional file

Additional file 1:
**SPIRIT (Standard Protocol Items: Recommendations for Interventional Trials) checklist (.doc).** Completed SPIRIT 2013 checklist of recommended items to address in a clinical trial protocol and related documents. (DOC 120 kb)

## Abbreviations

CALIS-PV: Children’s Anxiety Life Interference Scale – Preschool Version
DSM-IV: Diagnostic and Statistical Manual of Mental Disorders, 4th Edition
K10: Kessler 10 Psychological Distress Scale
OAPA: Online Assessment of Preschool Anxiety
OI/P: Over-Involved/Protective parenting scale
PAS-R: Revised Preschool Anxiety Scale
RCT: Randomised controlled trial
SDQ-ES: Strengths and Difficulties Questionnaire-Emotional Symptoms subscale
